# Effects of Consuming Ultraviolet Light-Exposed Mushrooms on Self-Reported Indices of Brain Health and Performance-Based Cognition in Middle-Aged and Older Adults

**DOI:** 10.3390/foods14183148

**Published:** 2025-09-09

**Authors:** Emily S. Glover, Skye C. Napolitano, Luz M. Comboni, James C. Fleet, Matthew R. Olson, Dan Foti, Wayne W. Campbell

**Affiliations:** 1 Department of Nutrition Science, Purdue University, West Lafayette, IN 47907, USA; hodson7@purdue.edu (E.S.G.); lcomboni@purdue.edu (L.M.C.); 2 Department of Psychological Sciences, Purdue University, West Lafayette, IN 47907, USA; snapolit@purdue.edu (S.C.N.); foti@purdue.edu (D.F.); 3 Department of Nutritional Sciences, The University of Texas at Austin, Austin, TX 78712, USA; james.fleet@austin.utexas.edu; 4 Department of Biological Sciences, Purdue University, West Lafayette, IN 47907, USA; olson126@purdue.edu

**Keywords:** mushrooms, vitamin D, brain health, depression, anxiety, mood, cognition, well-being

## Abstract

**Objectives**: Accumulating clinical evidence from experimental and observational studies with humans suggests that edible mushrooms may have beneficial effects on markers of brain health. This study examined the effects of daily consumption of fresh *Agaricus bisporus* (cremini mushrooms) exposed to ultraviolet (UV) light on indices of anxiety, depression, mood, cognitive function, and well-being in middle-aged and older adults. **Methods**: Over a 6-week period, adults (*n* = 41 (19 M/22 F), age 43 ± 11 y; BMI 29.8 ± 5.9 kg/m^2^, mean ± SD) without severe depression, cardiovascular disease, or Type 2 Diabetes consumed two daily servings (168 g/d wet weight) of cremini mushrooms intended to provide 400 IU/serving (800 IU/d) of vitamin D_2_ (*n* = 20) or 2 tsp/d of breadcrumbs (control, *n* = 21). Assessments conducted at baseline and week 6 included General Anxiety Disorder-7 (GAD-7), Beck’s Depression Inventory (BDI-II), Patient Health Questionnaire (PHQ-9), Repeatable Battery for the Assessment of Neuropsychological Status (RBANS), Profile of Mood States (POMS), and Medical Outcomes Study 36-Item Short-Form Health Survey Version 2 (SF36v2). **Results**: Consuming UV light-exposed mushrooms did not improve brain health outcomes. Independent of mushroom consumption, over time, there were improvements in immediate memory (RBANS), language (RBANS), and depression (BDI-II and PHQ-9). **Conclusions**: There were no differences observed between groups in the investigated indices of brain health. However, improvements over time were observed in Beck’s Depression Inventory and the Immediate Memory and Language domains in the RBANS, independent of mushroom consumption. Overall, consuming 2 servings/d of UV-exposed mushrooms for six weeks may not improve indices of brain health.

## 1. Introduction

The overarching concept of brain health includes numerous functions, such as anxiety, depression, mood, cognition, and well-being. Deterioration of brain health contributes to the development of psychiatric conditions and cognitive decline, which can greatly impact quality of life. Emerging research suggests mushrooms, unique in nutritional value, may be important modulators in the prevention of various brain health diseases [1,2,3,4,5,6].

Mushrooms, identified as vegetable but biologically classified as fungi, have many brain health-promoting compounds [7]. Mushrooms contain significant amounts of L-ergothioneine and glutathione, known for their protective effects against oxidative stress and beta-amyloid plaque deposition in the brain—key biomarkers for cognitive impairment [8,9,10]. The presence of a highly specific transporter, called Ergothioneine Transporter (ETT, previously named Organic Cation Transporter N1) in the blood brain barrier suggests the importance of ergothioneine in the brain’s cellular pathways [11]. Furthermore, older adults most at risk for declining brain health exhibit decreased plasma ergothioneine concentrations, with even greater reductions in those age-matched with mild cognitive impairment [12,13]. Similarly, older patients with cognitive impairment have decreased levels of glutathione compared to healthy controls [14]. Research indicates that many bioactive compounds present in mushrooms may mitigate declines in brain health, but research investigating the effects of whole, dietary mushrooms is limited.

Some epidemiological studies and randomized controlled trials suggest a potential association or causal link between mushroom consumption, either as a supplement or part of a healthy diet, and brain health. Findings from epidemiological studies indicate improvements in depressive symptoms and decreases in mild cognitive impairment in Asian individuals who self-reported >30 g serving and >2 servings/week, respectively, of any variety of mushroom as part of a health-promoting diet [15,16]. However, few of the epidemiological studies report mushrooms as a separately quantifiable component of a healthy diet. Conversely, researchers who conducted clinical trials report more mixed results, due to the varying intervention durations and mushroom types. In one study, Japanese participants with mild cognitive impairment demonstrated higher scores on cognitive function scales compared to the placebo group after consuming *Hericium erinaceus* (lion’s mane mushroom) tablets [17]. In another clinical trial, older adults consuming lion’s mane mushroom supplements exhibited improvements in cognitive function scores compared to the control [18]. All intervention studies [19]—except for one study including whole mushrooms when participants adopted a healthy Mediterranean dietary pattern [20]—assessed mushrooms in the form of an extract, dried powder, capsule/tablet, or a separate food formulated with mushroom powder.

In addition to their many bioactive compounds, mushrooms contain ergosterol, a predominant sterol in fungal cell membranes that undergoes photolysis upon ultraviolet (UV) light exposure, converting into ergocalciferol (vitamin D_2_). Besides non-fortified foods, mushrooms are the only naturally sufficient non-animal-derived source of vitamin D. Several systematic reviews [21,22] and individual studies [23,24] report vitamin D’s potential in reducing depressive symptoms, associating lower vitamin D with higher dementia risk along with decreased total brain volume. Highlighting the potential significance of pairing vitamin D with mushrooms, several studies have shown that UVB light-irradiated mushrooms are equally effective as vitamin D_2_ supplements in improving vitamin D status in humans [25,26]. To our knowledge, only one clinical trial [27] has assessed the effects of UV light-exposed mushrooms on brain health, with null findings after six months for changes in cognitive function or mood in older adults after consumption of capsules of UV light-exposed mushroom powder.

The purpose of this research was to assess the effects of consuming UV light-exposed mushrooms on indices of perceived anxiety, depression, mood, cognition, and well-being. While vitamin D_2_ content was a component of the intervention, the study was not a vitamin D-centric study. To our knowledge, the present study is among the first randomized placebo-controlled studies examining the effects of fresh, whole UV light-exposed mushrooms on mood- and cognitive-related outcomes in humans. The current study is exploratory due to the limited amount of human research assessing these outcomes.

## 2. Materials and Methods

### 2.1. Experimental Design

**Figure 1:**
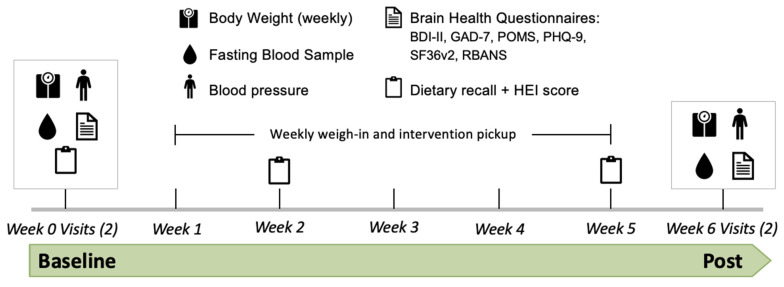
Six-week study design with outcomes assessed at baseline (week 0) and week 6. Assessments included Beck’s Depression Inventory (BDI-II), General Anxiety Disorder-7 (GAD-7), Profile of Mood States Short-Form (POMS-SF), Patient Health Questionnaire-9 (PHQ-9), Medical Outcomes Study 36-Item Short Form Health Survey Version 2 (SF36v2), and Repeatable Battery for the Assessment of Neuropsychological Status (RBANS). HEI = healthy eating index.

This study employed a randomized parallel design with 41 adults (19 M/22 F, age 43 ± 11 y; BMI 29.8 ± 5.9 kg/m^2^, mean ± SD) without diagnosed morbidities. While continuing to consume their usual, self-chosen diets, participants randomized into the Mushroom group consumed fresh UV light-exposed *Agaricus bisporus* (cremini mushrooms; Monterey Mushrooms, Princeton, IL, USA and Madisonville, TX, USA), and participants randomized in the Control group consumed commercial breadcrumbs. Brain health outcomes—anxiety, depression, mood, cognition, and well-being—were assessed at baseline (week 0) and post (week 6) (Figure 1). To control for seasonal fluctuations in endogenous vitamin D_3_ synthesis, data collection occurred between late fall and winter.

Prior to participant recruitment, the Purdue University Institutional Review Board approved the study protocol (IRB 2022-750), and it was registered at Clinicaltrials.gov (NCT05559112). Participants were asked to discontinue vitamin D supplements prior to starting the intervention and to maintain habitual dietary intakes and levels of physical activity during the intervention period. They provided written informed consent prior to starting the study and were financially compensated for their time.

### 2.2. Participant Inclusion Criteria

Male and female volunteers (30–69 y) with overweight or obesity (25.0–34.9 kg/m^2^), were recruited from West Lafayette and Lafayette, IN, and surrounding regions in the United States of America. Additional inclusion criteria included: total cholesterol < 240 mg/dL; low-density lipoprotein cholesterol (LDL) < 160 mg/dL; triglycerides < 300 mg/dL; fasting glucose < 110 mg/d; systolic/diastolic blood pressure < 140/90 mmHg; body weight stable for 3 months prior; medication use stable for 6 months prior; willing to refrain from taking vitamin D supplements or any supplements containing vitamin D; no history of bariatric surgery; consuming an unrestricted diet; not extremely or severely depressed (Beck’s Depression Inventory (BDI-II) Score < 30); non-smoking; non-diabetic; not acutely ill; females not pregnant or lactating; participants must agree to provide blood samples for at least one month prior to, during, and one month after the study; agree not to travel to sunny locations during the study period; and agree to forego any tanning bed or other tanning procedures during the study. Participants were required to be willing and able to consume mushrooms and travel to testing facilities. Prospective screeners who had been consuming doses of vitamin D greater than the Recommended Dietary Allowance (600 IU/d) but less than 3000 IU/d were asked to discontinue vitamin D supplementation for 4 weeks prior to beginning the study. Individuals consuming >3000 IU/d were not eligible for the study. Eligible participants were randomly assigned to consume either mushrooms or breadcrumbs using a pre-generated Excel randomization list based on ID order at study entry.

Research coordinators used a variety of recruitment strategies, including physical fliers, announcements on Purdue University’s daily email newsletter, and a partnership with Trialfacts, a company based in Melbourne, Australia, that specializes in participant recruitment services and advertisement. Additionally, consented individuals from previous studies in a participant database were also asked about potential interest.

### 2.3. Study Intervention and Dietary Assessment

During the intervention, participants in both groups consumed their habitual, self-chosen diets. Participants randomized to the Mushroom group were instructed to consume fresh mushrooms (168 g/d, 7 d/wk) intended to contain 400 IU/serving (800 IU/d) vitamin D_2_. They were given a kitchen scale along with instructions regarding mushroom preparation (raw, quick-sauteed, microwaved, or boiled), handling and storage, and how to identify if the mushrooms had spoiled. We used commercially available UV light-exposed cremini mushrooms grown in the United States; thus, we relied on the labeling of the commercial products for their vitamin D2 content. While participants were consuming mushrooms, we periodically froze mushroom samples (*n* = 20), which were batched and sent to Heartland Assays, Ames, IA, for vitamin D2 analysis using liquid chromatography-tandem mass spectrometry (LC/MS/MS). Among the 20 participants in the mushroom group, the average vitamin D2 intake during their six-week intervention periods was 169 ± 119 ng/g, which equates to 566 IU/84 g serving, and 1132 IU/d. Participants randomized to the Control group were instructed to consume 1 tsp (~2.3 g) breadcrumbs twice daily, mixed into any meal of their choice. They were also given separate instructions outlining the daily amounts to consume and storage instructions. Participants in the Control group were not made aware of the kind of food they were consuming, but participants in the Mushroom group were aware of their assignment due to the nature of the intervention. Participants were discouraged from sharing details of their intervention with other participants. They began consuming their assigned intervention the day after completing baseline testing.

Self-reported adherence to consuming the interventions was assessed using data from an online Qualtrics survey participants completed daily. In this survey, participants were asked whether they consumed their required amounts for the day and prompted to answer additional questions helpful for the researchers (i.e., if the participant was experiencing an illness, upset stomach, gassy symptoms, medication changes, etc.). Participants had consistent communication with the study coordinators through email conversations and weekly weigh-ins when they picked up their mushrooms or breadcrumbs.

The Automated Self-Administered 24-h Dietary Assessment Tool (ASA24; version 2022, developed by the National Cancer Institute of the National Institute of Health) was used to estimate their daily food consumption. Each participant completed 1 assessment at baseline and the remaining 2 assessments on nonconsecutive random weekdays and weekend days during the intervention. Dietary intake data were used to calculate participants’ Healthy Eating Index (HEI) scores to assess diet quality and consistency during the intervention and to demonstrate that our participant’s dietary patterns were generally consistent with those of the broader US population.

### 2.4. Clinical Assessments

For baseline and week-6 testing visits, participants came to the Purdue University clinical research facility following a 10 h overnight fast. Participants first weighed themselves in a private room with minimal clothing items to their comfort level to obtain the most accurate measurement. Participant’s heights were also measured at baseline and week 6 visits. Body weight and height were used to calculate body mass index (BMI, kg/m^2^). Participants then rested for 15 min in a seated position before blood pressure was measured, and a fasting blood sample was obtained and sent to Heartland Assays, Ames, IA, USA, for 25OHD_2_, 25OHD_3_, and total 25OHD analysis for via tandem liquid chromatography mass spectrometry (LC/MS/MS).

Assessments included General Anxiety Disorder-7 (GAD-7), Beck’s Depression Inventory (BDI-II), Patient Health Questionnaire-9 (PHQ-9), Profile of Mood States Short-Form (POMS-SF), Repeatable Battery for the Assessment of Neuropsychological Status (RBANS), and Medical Outcomes Study 36-Item Short Form Health Survey Version 2 (SF36v2).

#### 2.4.1. Assessment of Symptoms of Generalized Anxiety Disorder

Generalized anxiety disorder-7, a 7-item measure, was utilized to assess the symptoms of generalized anxiety disorder. The participant was asked to respond to each item to indicate the severity of potential symptoms over the previous 2 weeks [28]. The GAD-7 has good reliability, criterion, construct, factorial, and validity [29]. Thus, GAD-7 effectively evaluates significant symptoms of generalized anxiety in clinical practice and research [29].

#### 2.4.2. Assessment of Symptoms of Depression

Participants completed two measures for assessing symptoms of depression—BDI and PHQ-9. The BDI is a 21-item self-report inventory measuring the intensity of depression symptoms over the previous 2 weeks [30]. The BDI shows high reliability, ability to distinguish between depressed vs non-depressed subjects, and improved structural validity [31].

Supplementary to the BDI, the PHQ-9 is a 9-item self-reported measure for major depressive disorder over the previous 2 weeks and asks individuals how disrupting symptoms are to their daily functioning. The PHQ-9 is deemed a valid measure of depression severity with high reliability [32].

#### 2.4.3. Assessment of Mood

Participants’ moods were evaluated using the Profile of Mood States (POMS), a 37-item self-report survey that measures Total Mood Disturbance with 6 subscales (depression, vigor, confusion, tension, fatigue, and anger). Mood-related adjectives were presented and participants indicated the severity to which adjectives describe themselves during the past week. Ratings are collected using a Likert-type scale ranging from “0-not at all” to “4-Extremely”. The POMS is a widely used tool for assessing psychological distress [33].

#### 2.4.4. Assessment of Perceived Quality of Life

Self-perceived health status was assessed using the self-reported Medical Outcomes Study 36-item Short-Form Health Survey (SF-36v2). Participants rated their level of agreement with various health-related statements. The SF-36v2 scoring is based on 8 scales: physical functioning, role limitations due to physical health, role limitations due to physical problems, energy/fatigue, emotional well-being, social functioning, pain, and general health.

#### 2.4.5. Neuropsychological Assessment

The Repeatable Battery for the Assessment of Neuropsychological Status (RBANS) Update, Forms A and B, was used to assess cognitive function [34]. During this approximate 30 min assessment, participants completed 12 sub-tests assessing different domains of neuropsychological status (e.g., immediate and delayed memory, visuospatial awareness, attention, and language). The RBANS offers multiple versions, allowing participants to perform tasks with similar formats across visits, while the specific items and words differ each time. For this reason, this assessment was ideal for clinical trials with different timepoints, and the participants completed a different version at baseline and post visits, matched for difficulty. The RBANS has been validated with adequate diagnostic accuracy across a diverse selection of populations [35].

### 2.5. Statistical Analysis

All outcome data were entered independently twice and cross-checked. Data were analyzed according to a pre-specified plan, ensuring analysts remained blinded to the intervention status until all analyses were finalized. Descriptive statistics and bivariate associations among study variables were examined using SPSS (v. 29, IBM Corp., Armonk, NY, USA). Assumptions of linearity, homoscedasticity, and normality were assessed, and no violations were detected in any of the analyses. The primary analyses were performed as multiple two-way repeated measures analyses of covariance (ANCOVA) to determine whether mushroom consumption affected changes in brain and metabolic health outcomes. Sex was included as a covariate in all models due to its potential influence on the measured outcomes [36]. Although BMI was initially included as a covariate, it did not change significantly throughout the study and was therefore excluded from the final models to enhance efficiency and simplicity. HEI scores were included to characterize diet quality in the sample but were not included as covariates in the models, as diet quality was not conceptualized as a confounder or mediator in the associations of interest. No a priori power analysis was conducted, as this was an exploratory study utilizing secondary analyses of existing data.

## 3. Results

### 3.1. Participants

**Figure 2:**
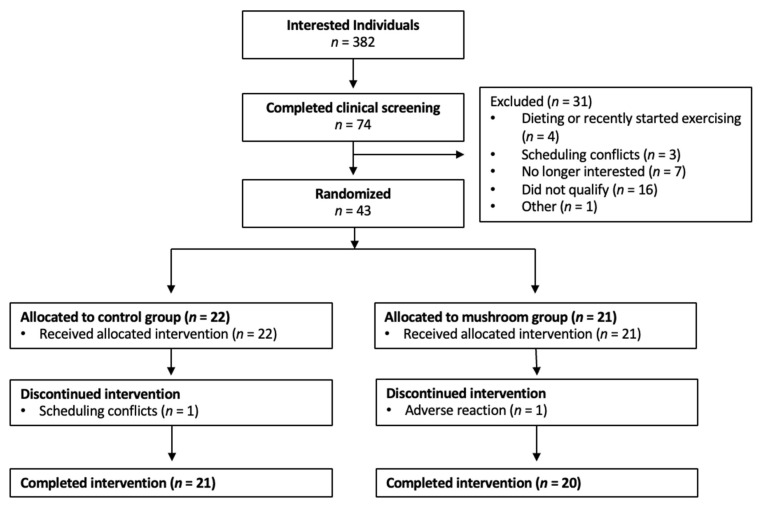
CONSORT participant flow diagram.

Study coordinators were in contact with 382 interested individuals during the clinical testing period (late autumn to late winter 2022–2023 and 2023–2024). Of the 382 individuals, 43 people were randomized to either Mushroom (*n* = 21) or Control (*n* = 22) groups. Forty-one participants completed the intervention as detailed in Figure 2. At baseline, most participants had normal depressive characteristics (*n* = 35, 85%) as measured by the BDI questionnaire (Table 1). Five participants were characterized as having mild mood disturbance and one with moderate depression symptoms. There were no differences between groups at baseline.

### 3.2. Dietary Assessment and Adherence to the Dietary Intervention

Thirty-nine participants completed the self-reported ASA24 food logs for calculating HEI-2015 scores. Mean HEI scores were calculated for each individual group and combined at baseline, during the intervention, and overall (Table 2). There were no differences between groups at baseline or during the intervention.

Dietary adherence to the intervention calculated based on the number of days participants self-reported adherence or non-adherence over the number of days they were in the study. The average adherence to consuming the assigned interventions across all participants was 97% (96.6% in the Mushroom group and 97.7% in the Control group).

### 3.3. Anxiety and Depression

There were no differential changes in anxiety scores as assessed by GAD-7 after consumption of the mushrooms in any of the analyses. Independent of mushroom consumption, Beck’s Depression Inventory scores decreased (improved) over time (*p* = 0.019). There were no changes in the other measure for depression (PHQ-9) (Table 3).

### 3.4. Mood

There were no changes in any of the mood outcomes (depression, vigor, confusion, tension, fatigue, and anger) assessed via the Profile of Mood States (Table 4).

### 3.5. Perceived Quality of Life

There were no changes in any of the wellbeing outcomes (physical functioning, role limitations due to physical or emotional problems, energy/fatigue, emotional wellbeing, and general health) assessed via the Medical Outcomes Study 36-Item Short Form Health Survey Version 2 (SF-36v2) (Table 5).

### 3.6. Neuropsychological Function

There were no changes between groups in any of the cognitive outcomes (immediate memory, visuospatial construction, language, attention, and delayed memory) assessed via RBANS (Table 6). Scores improved in both groups from base to week 6 in immediate memory (*p* < 0.001) and language (*p* = 0.035) domains.

## 4. Discussion

The objective of the present study was to assess the effects of incorporating UV light-exposed whole mushrooms into the diet, as a food-based intervention, compared to a non-mushroom control. While vitamin D_2_ content was a component of the intervention, the study was not intended as a vitamin D-centric study. To our knowledge, the present study was the first randomized controlled trial to investigate the effects of dietary UV light-exposed mushrooms on indices of brain health, with only one other published study including whole mushrooms not exposed to UV light [20], and another including UV light-exposed mushrooms in a powdered capsule form [27]. Our findings suggest that consuming 2 servings/d of UV light-exposed *A. bisporus* (cremini) mushrooms for 6 weeks does not improve indices of anxiety, depression, mood, cognitive function, and well-being in non-clinical middle-aged and older adults.

The only other study, to our knowledge, investigating the effects of vitamin D-enriched mushrooms (100 mg white button capsules with 300 IU vitamin D_2_ each, 2 x/d) on brain health, compared to standard mushroom capsules, vitamin D_3_ supplements (600 IU), and placebo, reported no changes in mood or cognitive outcomes in non-clinical elderly adults after 24 weeks [27]. This study was also conducted during the winter months to avoid confounding effects of vitamin D_3_ synthesis from the sun. While this study reported numerous similarities to the current study, some key differences are worth noting. These differences include variations in study duration (6 vs. 24 weeks), population (middle-aged and older adults vs older adults), and nature of the intervention (whole, fresh cremini mushrooms vs dried, white button mushroom capsules). Our findings, indicating that UV light-exposed mushrooms do not improve mood and well-being outcomes, are partly inconsistent with previous research. Existing research indicates consuming lion’s mane in the form of capsules [37,38] or baked into cookies [39] improves anxiety, stress, and depression. Researchers from two studies suggest that consuming 500 mg (3 x/d) lion’s mane capsules for 8 weeks improves depressive and anxious mood disorders in individuals with mood and/or binge-eating disorders [38], and 600 mg (3 x/d) lion’s mane capsules for 4 weeks improves stress-induced mood disturbances in non-clinical young adults [37]. Furthermore, evidence suggests that cookies containing dried lion’s mane powder (2 g/d, 0.5 g/cookie) reduces depression and anxiety after 4 weeks in non-clinical female adults [39]. Similar to our findings, other studies report no changes in mood or well-being outcomes [20,40]. Questionnaires utilized in these studies include Positive and Negative Affect Scale (PANAS), Zung’s Depression and Anxiety Self-Assessment Scales, Symptom Checklist-90 (SCL-90), the 21-item Depression, Anxiety, and Stress Scale (DASS-21), the General Happiness Scale, and Perceived Stress Scale (PSS), with only one study implementing the same measurements as the current study [20]. Results from interventional studies assessing mood outcomes vary in study design but suggest a reduction in depressive symptoms after consuming a form of lion’s mane mushrooms. Importantly, comparing the results of our work to other published findings proves difficult due to the variability in study designs, especially the differences in populations tested and the doses/administration of the interventions.

The current study indicated no improvements in cognitive function after consuming UV light-exposed mushrooms, which is partly inconsistent with previous research. Researchers from one study indicated that consumption of 5 g muffins (2 x/d) formulated with lion’s mane powder does not influence cognitive function in non-clinical young adults [41]. Similarly, results from another study suggested that consuming 1.8 g of lion’s mane powder capsules daily for four weeks had no impact on cognitive function, as measured by the Computerized Mental Performance Assessment System (COMPASS), in non-clinical adults [37]. Conversely, separate researchers reported their findings that consuming 0.8 g (4 x/d) lion’s mane supplements for 12 weeks improved cognitive function (assessed via Mini Mental State Examination, MMSE) in non-clinical adults aged 55–65 years [18]. Similar to their findings, another study reported that consuming four 250-mg lion’s mane tablets (3 x/d) for 16 weeks improved cognition (assessed via the Revised Hasegawa Dementia Scale, HDS-R) in 50–80-year-old adults with mild cognitive impairment [17]. Intervention studies report mixed results regarding cognitive outcomes, likely due to the heterogeneity of the study designs. Broadly, cognitive improvements may be less likely to be observed in young adults compared to middle-aged and older adults, and non-clinical adults may have different responses compared to adults with cognitive impairment. Furthermore, as discussed previously, variations in the types of assessments used and doses of the interventions create difficulty in comparing the results among studies.

Strengths of the present study include the use of a randomized, controlled study design. There was high adherence to the intervention (97% self-reported mean adherence to consuming the assigned intervention) and low drop-out percentage (<5%). Our study design considered confounding factors from participants’ daily living, including maintaining habitual exercise and diet and discontinuing vitamin D supplements. If participants were taking a high dose of vitamin D (>600 IU and <3000 IU) prior to joining the study, they underwent a brief washout period (~4 weeks) before beginning the intervention. Additional steps were taken to reduce risk of bias, including de-identification of data, double data entry, and cross-checking. Statistical analysts were blinded to group assignments. A limitation of the present study was the inability to blind both participants in the intervention group and research coordinators due to the nature of the intervention. However, participants in the Control group remained blinded, as the intervention could plausibly be delivered in powdered form.

Future studies would be helpful to investigate the effects of mushrooms in individuals with declining cognition or diagnosed with cognitive impairment. Only 3 mushroom studies [17,42,43] have been published with this population, with differing mushroom types (*Ganoderma lucidum* [42] and lion’s mane [17,43]) and mixed results. Our sample of non-clinical individuals may have limited the intervention from having observable effects. Additionally, a 6-week intervention may not be long enough to observe changes in brain health with this dosage of mushrooms, particularly in a non-clinical population. We chose this time frame to stay within the winter season, and the heterogeneity in study designs and mushroom types offered little guidance. Therefore, future research may employ a longer study duration to observe long-term effects in brain health. Moreover, cremini mushrooms may not contain adequate amounts of certain bioactive compounds present in other mushrooms that may have a greater beneficial impact on brain health. In terms of UV light-exposed mushrooms, only the present study and one other [27] report its effects on brain health outcomes. Both studies report similar results, however, future studies could examine these effects in clinical populations.

## 5. Conclusions

In conclusion, UV light-exposed cremini mushrooms consumed for 6 weeks did not influence anxiety, depression, mood, cognitive function, or well-being in healthy (non-clinical) middle-aged and older adults. To our knowledge, this is the first randomized controlled trial to assess the effects of whole, UV light-exposed mushrooms on brain health outcomes, adding valuable evidence to a limited body of research. Despite strong adherence and a controlled study design, the lack of observed benefits suggests that UV light-exposed mushrooms may not confer additional cognitive or mood-related advantages in already healthy populations over a short intervention period.

## Figures and Tables

**Table 1:** Baseline demographic and depression characteristics.

Demographic Characteristics	Control (*n* = 21)	Mushroom (*n* = 20)	Total (*n* = 41)
Age (y)	42.2 ± 11.2	44.0 ± 11.1	43.1 ± 11.1
Female, *n* (%)	12 (57)	10 (50)	22 (54)
White, *n* (%)	11 (52)	15 (75)	26 (63)
Hispanic or Latino, *n* (%)	5 (24)	2 (10)	7 (17)
Asian, *n* (%)	4 (19)	3 (15)	7 (17)
Black, *n* (%)	1 (5)	1 (5)	2 (5)
Other (not specified/not reported), *n* (%)	5 (24)	1 (5)	6 (15)
Weight (kg)	82.3 ± 10.0	87.3 ± 15.6	84.7 ± 13.1
BMI (kg/m^2^)	29.9 ± 7.9	29.7 ± 2.7	29.8 ± 5.9
Fasted clinical characteristics			
25(OH)D_3_ (ng/mL)	23.3 ± 7.5	21.7 ± 5.6	22.5 ± 6.6
25(OH)D_2_ (ng/mL)	0.2 ± 0.9	0.0 ± 0.0	0.1 ± 0.6
* Total 25OHD (ng/mL)	23.5 ± 7.8	21.7 ± 5.6	22.6 ± 6.8
BUN (mg/dL)	15.1 ± 5.0	12.3 ± 3.0	15.1 ± 4.3
Creatinine (mg/dL)	0.9 ± 0.2	0.8 ± 0.1	0.8 ± 0.1
BUN/Creatinine ratio	15.1 ± 5.0	15.1 ± 3.4	15.1 ± 4.3
eGFR (mL/min/1.73 m^2^)	98.1 ± 13.0	101.6 ± 13.3	99.7 ± 13.1
ALT (U/L)	19.1 ± 13.7	18.6 ± 9.4	18.9 ± 11.7
AST (U/L)	19.6 ± 7.3	20.1 ± 5.2	19.8 ± 6.3
Depression Characteristics			
Beck’s Depression Inventory (0–63)	5.14 ± 7.04	5.25 ± 4.79	5.20 ± 6.0
Levels of depression, *n* (%)			
Normal (1–10)	18 (86)	17 (85)	35 (85)
Mild Mood Disturbance (11–16)	2 (10)	3 (15)	5 (12)
Borderline Clinical Depression (17–20)	0 (0)	0 (0)	0 (0)
Moderate Depression (21–30)	1 (5)	0 (0)	1 (2)
Severe Depression (31–40)	0 (0)	0 (0)	0 (0)
Extreme Depression (>40)	0 (0)	0 (0)	0 (0)

Data are means ± SD. Differences at baseline were not statistically significant. * Total 25(OH)D = 25(OH)D_3_ + 25(OH)D_2_. Abbreviations: ALT, alanine transaminase; AST, aspartate transferase; BUN, blood urea nitrogen; eGFR, estimated glomerular filtration rate.

**Table 2:** Healthy Eating Index scores for participants in each group at each time point.

Timepoint	Mushroom	Control	Mushroom + Control	*p*-Value
Baseline	50 ± 15.3	55 ± 15.3	53 ± 15.4	0.288
Intervention	50 ± 10.9	52 ± 14.1	51 ± 12.5	0.730
Baseline + Intervention	50 ± 10.3	53 ± 12.1	52 ± 11.2	0.475

Values are means ± SD. Heathy Eating Index scores can range from 0 to 100 arbitrary units, with a higher score indicating a healthier dietary pattern.

**Table 3:** Changes in indices of perceived anxiety and depression after 6 weeks of consuming UV light-exposed mushrooms or control.

	Control (*n* = 21)			Mushroom (*n* = 20)			*p*-Values	
Outcome	Baseline	Week 6	Change	Baseline	Week 6	Change	Time	Time × Group
Generalized Anxiety Disorder-7 (0–21)	3.10 ± 0.92	2.33 ± 0.57	−0.65 ± 0.72	2.70 ± 0.67	2.30 ± 0.79	−0.40 ± 0.50	0.200	0.698
Beck’s Depression Inventory (0–63)	5.14 ± 1.54	4.90 ± 1.43	−0.24 ± 0.47	5.25 ± 1.07	3.40 ± 0.82	−1.85 ± 0.75	0.019 *	0.092
Patient Health Questionnaire-9 (0–27)	3.48 ± 0.70	3.10 ± 0.77	−0.38 ± 0.51	3.45 ± 0.70	3.15 ± 0.81	−0.30 ± 0.51	0.277	0.796

Results are the marginal means ± SEM (*n* = 41 total). Change values were calculated by subtracting baseline values from week 6 values. * Indicates statistical significance at the *p* < 0.05 level.

**Table 4:** Changes in mood measured by the Profile of Mood States after 6 weeks of consuming UV light-exposed mushrooms or control.

	Control (*n* = 21)			Mushroom (*n* = 20)			*p*-Values	
Mood	Baseline	Week 6	Change	Baseline	Week 6	Change	Time	Time × Group
Depression (0–32)	1.10 ± 0.44	1.76 ± 0.54	0.67 ± 0.52	1.35 ± 0.42	1.70 ± 0.82	0.35 ± 0.89	0.389	0.853
Vigor (0–24)	10.3 ± 1.43	11.8 ± 1.44	1.43 ± 1.07	10.1 ± 1.39	10.0 ± 1.25	−0.63 ± 0.98	0.484	0.100
Confusion (0–20)	1.71 ± 0.40	1.95 ± 0.41	0.24 ± 0.31	2.20 ± 0.47	2.15 ± 0.51	−0.05 ± 0.46	0.685	0.560
Tension (0–24)	1.75 ± 0.48	2.10 ± 0.55	0.45 ± 0.57	1.45 ± 0.41	2.10 ± 0.79	0.65 ± 0.37	0.251	0.821
Fatigue (0–20)	2.90 ± 0.74	3.57 ± 0.82	0.70 ± 0.38	3.45 ± 0.76	3.35 ± 0.69	−0.10 ± 0.45	0.293	0.482
Anger (0–48)	0.60 ± 0.27	0.95 ± 0.43	0.35 ± 0.52	0.75 ± 0.37	1.80 ± 0.70	1.05 ± 0.71	0.145	0.373

Results are the marginal means ± SEM (*n* = 41 total). Change values were calculated by subtracting baseline values from week 6 values.

**Table 5:** Changes in perceived quality of life measured by SF-36v2 after 6 weeks of consuming UV light-exposed mushrooms or control.

	Control (*n* = 21)			Mushroom (*n* = 20)			*p*-Values	
SF-36v2 Scale (0–100)	Baseline	Week 6	Change	Baseline	Week 6	Change	Time	Time × Group
Physical Functioning	91.0 ± 2.38	89.8 ± 2.70	−1.25 ± 2.40	91.0 ± 3.66	91.1 ± 2.95	0.53 ± 1.57	0.939	0.679
Physical RL	91.3 ± 4.54	88.1 ± 6.13	−3.75 ± 5.22	92.5 ± 5.47	92.5 ± 3.19	0.00 ± 6.28	0.757	0.757
Emotional RL	88.9 ± 5.79	80.9 ± 7.12	−7.94 ± 6.04	83.3 ± 6.17	75.0 ± 8.33	−8.33 ± 7.20	0.115	0.884
Energy/Fatigue	60.7 ± 4.49	60.7 ± 4.66	0.00 ± 2.60	63.5 ± 4.81	63.3 ± 5.35	−0.25 ± 2.53	0.864	0.751
Emotional Well-being	78.1 ± 3.62	78.1 ± 2.86	0.00 ± 2.19	78.8 ± 2.84	80.8 ± 3.33	2.00 ± 3.04	0.502	0.687
Social Functioning	91.7 ± 3.03	92.3 ± 3.17	0.60 ± 2.20	90.6 ± 2.99	91.9 ± 3.30	1.25 ± 3.50	0.584	0.960
Pain	79.9 ± 4.54	82.4 ± 4.23	2.50 ± 5.21	86.1 ± 3.87	87.8 ± 4.59	1.62 ± 2.36	0.550	0.957
General Health	72.8 ± 3.69	71.0 ± 3.56	−1.90 ± 2.79	72.0 ± 3.78	75.8 ± 2.91	3.75 ± 2.43	0.564	0.157

Results are the marginal means ± SEM (*n* = 41 total). Change values were calculated by subtracting baseline values from week 6 values.

**Table 6:** Changes in cognitive function measured by RBANS after 6 weeks of consuming UV light-exposed mushrooms or control.

	Control (*n* = 21)			Mushroom (*n* = 20)			*p*-Values	
Outcome	Baseline	Week 6	Change	Baseline	Week 6	Change	Time	Time × Group
Immediate Memory	103.8 ± 3.21	109.5 ± 4.17	6.76 ± 3.12	99.6 ± 2.81	108.0 ± 2.40	8.45 ± 2.66	<0.001 ***	0.616
Visuospatial/Constructional	84.4 ± 2.59	83.5 ± 3.18	−0.95 ± 2.63	88.5 ± 3.18	88.8 ± 3.34	0.30 ± 2.63	0.852	0.728
Language	98.6 ± 3.23	101.1 ± 3.68	2.52 ± 3.02	95.5 ± 3.34	101.9 ± 2.50	6.40 ± 2.73	0.035 *	0.339
Attention	103.8 ± 3.03	106.1 ± 3.63	2.29 ± 2.41	106.6 ± 3.68	110.6 ± 3.89	4.00 ± 2.61	0.074	0.705
Delayed Memory	98.6 ± 2.06	98.6 ± 2.99	0.00 ± 2.53	98.1 ± 1.50	100.2 ± 2.38	2.10 ± 1.90	0.610	0.437

Results are the marginal means ± SEM (*n* = 41 total). Change values were calculated by subtracting baseline values from week 6 values. * Indicates statistical significance at the *p* < 0.05 level; *** *p* < 0.0001.

## Data Availability

The original contributions presented in the study are included in the article. Further inquiries can be directed to the corresponding author.
